# The history of the Purine Club: a tribute to Prof. Geoffrey Burnstock

**DOI:** 10.1007/s11302-020-09749-4

**Published:** 2020-11-09

**Authors:** Maria P. Abbracchio

**Affiliations:** Department of Pharmaceutical Sciences, University of Milan, Via Balzaretti 9, 20133 and Via Festa del Perdono 7, 20122 Milan, Italy

**Keywords:** Purinergic signalling, Purine Club, Geoffrey Burnstock

## Abstract

The international purinergic scientific community has lost its pioneer. Geoffrey Burnstock, born on the 10th of May 1929 in London, died on the 2nd of June 2020, aged 91, in Melbourne (Australia). Geoff was one of the most highly regarded scientists of his generation. In the 1960s and 1970s, he developed a radical and somehow heretical new theory and opened an entire new field of science, signalling via extracellular nucleotides (the “purinergic theory”), which revolutionized our understanding of how cells communicate between each other. Initially, his unconventional theory found a lot of resistance in the scientific community. Once, one scientist even threatened to devote his entire life to disproving Burnstock’s theory. Undeterred, Geoff went further on, and continued to accumulate evidence in favour of his hypothesis, and led the field ever since. He struggled to attract new scientists to this new field of research and, in the early 1990s, due to new molecular biology techniques making it possible to isolate and identify cell surface receptors for ATP and its breakdown product adenosine, did evidence emerge that eventually convinced the doubters. The number of spontaneous obituaries and messages honouring Geoff’s memory that have appeared on specialized Journals and in the public press throughout the world since last June indicates that many people are clearly affected by his death. Besides being a rigorous, ethical and extremely brilliant scientist, Geoff was an extraordinary human being, always eager to collaborate and share data, never jealous of his findings and capable of learning things even from young people. He was known for his enthusiasm, empathy and ability to motivate young scientists and promote their careers. After the establishment of the Purine Club back in the 1990s, numerous Purine Club Chapters have been formed around the world with Geoff’s help and encouragement. He has obviously also been the inspirator and founder of our Journal, *Purinergic Signalling* (PUSI). For this reason, Charles Kennedy, the current Editor of the Journal, and myself thought that it might be nice to invite representatives from all known Purine Clubs to send a few notes to be published in PUSI on the history of their club and how Geoff inspired, aided or supported them. Here, I have collected all their contributions and I share with the entire purinergic community my personal memories on how the Purine Club was born and developed thanks to the invaluable mentoring of Geoffrey Burnstock. I apologize in advance if I am missing some information or forgot to mention somebody, and I strongly encourage all readers to submit memories and additional information that I shall gather for future writing. Keeping alive the history of how the field developed will be the best tribute that we can play to celebrate Geoff’s work along the years.

## The early days and the birth of the Purine Club

The history of the Purine Clubs dates back to the mid-1980s of the last century. Everything started in Italy. Simply based on the desire of sharing their common interest in the adenosine field, some Italian scientists started meeting regularly to exchange data and stimulate discussion in the field. The first very informal meeting was proposed by Giuliana Fassina, who had established her lab in Padua to study the effects of adenosine in the cardiovascular system; Flaminio Cattabeni, a neuroscientist and pharmacologist working at the University of Milan; and myself (then a young doctoral fellow at the same University). The two of us had originally developed an interest in adenosine as a follow-up to our studies on the long-term behavioural effects induced by the gestational administration of caffeine (one of adenosine’s natural antagonists). This first informal meeting was eventually organized in Florence in 1985, with the help of neuro-pharmacologists Giancarlo Pepeu and Felicita Pedata, who had a long history in studying adenosine effects in the brain. This initial meeting was followed by other informal gatherings that, in the subsequent years, attracted more and more Italian scientists from many different fields. Just to mention some, the group of Gloria Cristalli, a pharmaceutical chemist in Camerino, the group of pharmacologists led by Francesco Caciagli in Chieti, and a very strong group from Ferrara, composed by pharmacologist Pier Andrea Borea, medicinal chemist Pier Giovanni Baraldi, and Francesco Di Virgilio, a pathologist and cellular biologist who was actually the only one at those times working on ATP. These people already had strong international collaborations (e.g. with Ken Jacobson and Michail Sitkovsky at the National Institutes of Health in Bethesda, USA; Herbert Zimmermann in Germany; Luiz Belardinelli at University of Florida), constituting the basis of the future purinergic network that would have been so important in the subsequent years to spread the purinergic theory worldwide.

In 1987, I first met Geoffrey Burnstock in Pisa at a physiological meeting organized by the local National Research Council (CNR). For the first time, I was introduced to the wonders of ATP and was fascinated by Geoff’s infectious enthusiasm and ability to attract people to the field. I felt that something should be done to reunite under one single hat all the people working in the field of adenosine and ATP and shared this feeling with Geoff. He immediately agreed that people interested in adenosine and ATP were not talking to each other enough, and that research in this area would have enormously benefited from implementation of reciprocal interactions. After returning to Milan, I shared these thoughts with Flaminio, who agreed on the need to establish a non-profit scientific association aimed at gathering all the academic, industrial, preclinical and clinical researchers interested to the pathophysiology of purines.

To get a flavour of what was happening in the field at international level, in 1990, Felicita and myself decided to take part to one of the “International Conferences on Physiological and Regulatory Functions of Adenosine and Adenine Nucleotides” that were being held throughout the world every 4 years—the first of which had been held in Banff, Alberta, Canada, in 1978. In 1990 the conference was being held in Yamanaka, Japan. Out of approximately 120 participants, Felicita and I were actually the only two from Italy, and, together with another lady from Germany, the only women scientists (luckily, since then, many women scientists have entered the field and successfully contributed to it!). It was anyhow a great experience, both scientifically and from a social and human point of view. In Yamanaka, I approached Geoff to give him a letter written by Flaminio on the idea of the Purine Club. At that time, I did not know Geoff well and had not realized how “democratic” and open he was, so I felt that he would not probably give credit to a young kid like me, and that I needed to strengthen my idea with the formal engagement of a senior and reliable scientist like Flaminio. Geoff was obviously enthusiastic at this idea and immediately invited me to both discuss this issue and join his lab in London for a sabbatical leave—which I did later in 1992–1993. In Yamanaka, I also happened to see Ken Jacobson for the first time and to meet Nomi Burnstock, who struck me for her sharp intelligence and sense of humour and with whom I started a very nice friendship that grew a lot during my stay in London and in the subsequent years.

In 1991, Flaminio Cattabeni, Lina Puglisi and myself officially formalized the “Purine Club” in Milan, in front of a lawyer. The original Statutes of the Purine Club were inspired by Giuliana Fassina, based on her experience with the former Lipid Club, another Italian scientific, no profit, initiative to which she had personally contributed. Flaminio was the first elected President, with myself acting as a Councillor and Scientific Secretary. The first “Council” also included Giuliana Fassina, Mario Marzilli (a clinician), Antonio Lucacchini (a biochemist) and Giancarlo Pepeu.

We then immediately started organizing our first activity: the Purines 1992 meeting, to be held in Milan in June 1992 in the beautiful settings of the Ca’ Granda, the old site of the University of Milan. Geoff was extremely proactive in the organization of this meeting and gave us a lot of useful suggestions on the sessions to set up and on the people to invite. Since Geoff was already very well-known and appreciated worldwide, having him as one of the Presidents of the conference magically opened all doors, and this certainly contributed to the success of the meeting that witnessed the enthusiastic participation of more than 500 scientists from all over the world. Such a lively and informal scientific exchange fostered the Italian Purine Club to open internationally with the proposal (and establishment) of the National Purine Club Chapters**,** that were officially approved on the occasion of the “Adenosine and Adenine Nucleotides” meeting organized by Luiz Belardinelli in Philadelphia, USA, in 1994. That decision set the basis for the formal openings of the German, Spanish-Portuguese, Japanese, North American, UK, Danish, and, more recently, Brazilian, Australian, New Zealand, and Chinese Purine Club, for each of which we present a short story in the following sections.

## The Purines meetings and the Purine prizes

On the occasion of the Philadelphia meeting in 1994, it was also agreed by the scientific community to “merge” the new Purines series of meetings started in Milan in 1992 with the previous already ongoing “Adenosine and Adenine Nucleotides” conferences, with the aim of having one international meeting every 2 years easily recognizable by the name “Purines” followed by the year. On this basis, in the subsequent years, the following international conferences were organized:

- Purines 1996 (Milan, Italy)
- Purines 1998 (Ferrara, Italy)
- Purines 2000 (Madrid, Spain)
- Purines 2002 (Gold Coast, Australia)
- Purines 2004 (Chapel Hill, USA)
- Purines 2006 (Ferrara, Italy)
- Purines 2008 (Copenhagen, Denmark)
- Purines 2010 (Tarragona, Spain)
- Purines 2012 (Fukuoka, Japan)
- Purines 2014 (Bonn, Germany)
- Purines 2016 (Vancouver, Canada)
- Purines 2019 (Santiago de Compostela, Spain) that also coincided with the First European Purine Meeting

Besides these conferences, during the “Medicinal Chemistry and Pharmacology of Purinergic Receptors” workshop (Satellite of the 14th Camerino-Noordwijkerhout Symposium), held in Camerino (Italy) in 2003, it was agreed to start the series of the Joint Italian-German Purine Club meetings. These conferences have been regularly held in alternate years between the main International Purines Conference:

- 1st Joint Italian-German Purine Club Meeting 2005 (Chieti, Italy)
- 2nd Joint Italian-German Purine Club Meeting 2007 (Leipzig, Germany)
- 3rd Joint Italian-German Purine Club Meeting 2009 (Camerino, Italy)
- 4th Joint Italian-German Purine Club Meeting 2011 (Bonn, Germany)
- 5th Joint Italian-German Purine Club Meeting 2013 (Rimini, Italy)
- 6th Joint Italian-German Purine Club Meeting 2015 (Hamburg, Germany)

Additional congresses on purinergic transmission were also organized on specific topics and on volunteer basis, such as the 1st International Workshop on Nucleotides and their Receptors in the Immune System (held in Ferrara in 2000), the conference for the presentation of the prestigious Gold Medal of the University of Ferrara to Prof. Geoffrey Burnstock, held again in Ferrara in 2009, and the prestigious Ciba Foundation Symposia, strongly inspired by Geoff himself, held in London, England, in 1995 and in 2005.

Another important activity of the Purine Clubs that perfectly fits with their educational and scientific missions has been the establishment of prizes. The first prize (the Giuliana Fassina Prize) was established by the Italian Purine Club Chapter to honour the memory of Prof. Giuliana Fassina, one of the founders of the Purine Club, who had unfortunately died in the meantime. The Fassina Prize to distinguished scientists for extraordinary contributions to the purinergic field was first awarded to Dr. Kenneth A. Jacobson of The National Institutes of Health, Bethesda, USA, on the occasion of the Purines 1996 meeting in Milan. Since then, the prize has been awarded to Prof. Luiz Belardinelli (delivered on the occasion of the 8th International Symposium on Adenosine and Adenine Nucleotides held in Ferrara in 2006); to Prof. Peter Illes of the University of Leipzig, Germany (delivered to him in person on the occasion of the Joint German-Italian Purine Club meeting held in July 2011 in Bonn), to Prof. Pier Andrea Borea of the University of Ferrara, Italy, and to Prof. Herbert Zimmermann of the University of Frankfurt, Germany (delivered to both of them on the occasion of the Joint German-Italian Purine Club meeting held in September 2013 in Rimini), and, finally, to Prof. Flaminio Cattabeni (delivered to him in person on the occasion of the Congresso della Società Italiana di Farmacologia held in October 2015 in Naples).

The Burnstock Lecture was originally proposed in Chapel Hill by the North America Purine Club Chapter on the occasion of the Purines 2004 conference, when, during the opening ceremony of the conference, the organizers awarded it to Geoffrey Burnstock in recognition of his invaluable contribution to the field and invited him to deliver the first Burnstock Lecture. This came to Geoff as a very pleasant surprise and I believe that this has been one of the most rewarding events of his scientific (and personal) life. After that, several other scientists were invited to deliver the Burnstock Lecture, like Francesco Di Virgilio on the occasion of Purines 2014 in Bonn and Maria Abbracchio on the occasion of Purines 2019 in Santiago de Compostela.

The John Daly Lecture, in memory of the noted natural products chemist and pharmacologist in NIDDK who also greatly contributed to the purinergic field, was delivered only once in Bonn by Ken Jacobson during the Purines 2014 meeting.

## Our journal: *Purinergic Signalling* By Charles Kennedy, Glasgow, UK

Throughout the 1990s and into the 2000s, the number of research papers and reviews published that related to purinergic research began to rise substantially. This was in response particularly to the cloning of multiple P2X and P2Y receptors and the initial development of antagonists and later, subtype-selective compounds. These advances led to a rapid explosion of interest in purinergic signalling in a wide range of biological systems, such as the cardiovascular, respiratory, nervous, urogenital, musculoskeletal and gastrointestinal systems. In addition, changes in expression of receptors during development, ageing and disease could now be studied in more detail and the therapeutic potential of purinergic drugs investigated.

In response to this expansion of the field, Geoff Burnstock felt that it was time to launch a new journal that was devoted to purinergic signalling. His aim was to bring together the diverse molecular, physiological, biochemical, pharmacological and clinical studies of different systems and to encourage bridges to be built between basic science, clinical medicine and industrial development. Working together with a group of highly respected international leaders in the field and with Springer, he, therefore, created *Purinergic Signalling*.

The new journal was announced at the Purines 2004 Meeting in Chapel Hill and the inaugural issue was published in December 2004. It contained a series of reviews written by invited speakers at the Chapel Hill meeting. Since then, *Purinergic Signalling* has gone from strength to strength. The long-term contribution of Gillian Knight in assisting Geoff in the day-to-day running of the journal cannot be overestimated, and it continues to grow and develop and to be at the forefront of purinergic research. Early in 2020, Geoff asked Charles Kennedy to become Deputy Editor-in-Chief of *Purinergic Signalling*, and he was delighted to agree to do so. Charles assumed the day-to-day running of the journal once Geoff fell ill, and together with the excellent board of Associate Editors that Geoff had recruited, his aim is to ensure that *Purinergic Signalling* continues to be the natural home for reporting and discussing purinergic research.

## The Purine Club chapters

### The Italian Purine Club by Felicita Pedata, Florence, Italy

**Fig. 1:**
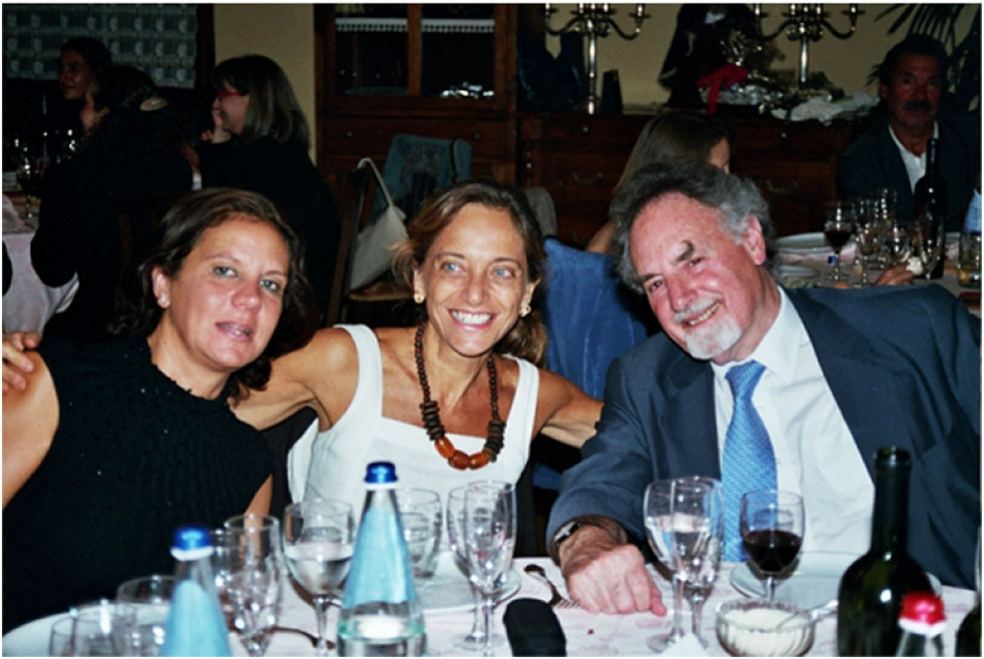
From left to right: Claudia Martini, Maria Abbracchio and Geoff Burnstock in the 2000s at one of the Italian Purine Club meetings

In January 2020, the Italian Purine Club had the privilege of conducting a public interview with Geoffrey Burnstock, who, as usual, showed himself to have a sharp intellect and the ability and sensitivity to share episodes and reflections of his life, and as a person who was passionate about his work and family. Italian purine researchers have had the honour to be pioneers in constituting the first Purine Club in the world at the time when the Burnstock’s hypothesis that ATP a neurotransmitter was beginning to be accepted (see also “The early days and the birth of the Purine Club” section). The Italian Purine Club has organized a number of international meetings and fostered the establishment of the National Purine Club Chapters (Fig. 1).

The Italian Purine Club now includes 100 members who are actively involved in Purine National and International meetings. Several travel grants have been raised to support participation of young researchers in purine meetings. A quarterly newsletter was recently established, taking advantage of the availability shown by young members to update the Club on recent publications related to the world of purines. Since 2005, very fruitful Joint Italian/German Purine Club meetings have been held, right up to the last one held in Rome in July 2017, when all members agreed that the purine field had grown large enough to include all European countries. The 1st European Congress on Purines in 2019 in Spain in Santiago De Compostela was welcomed with enthusiasm.

### The German Purine Club by Christa Muller, Bonn, Germany

**Fig. 2:**
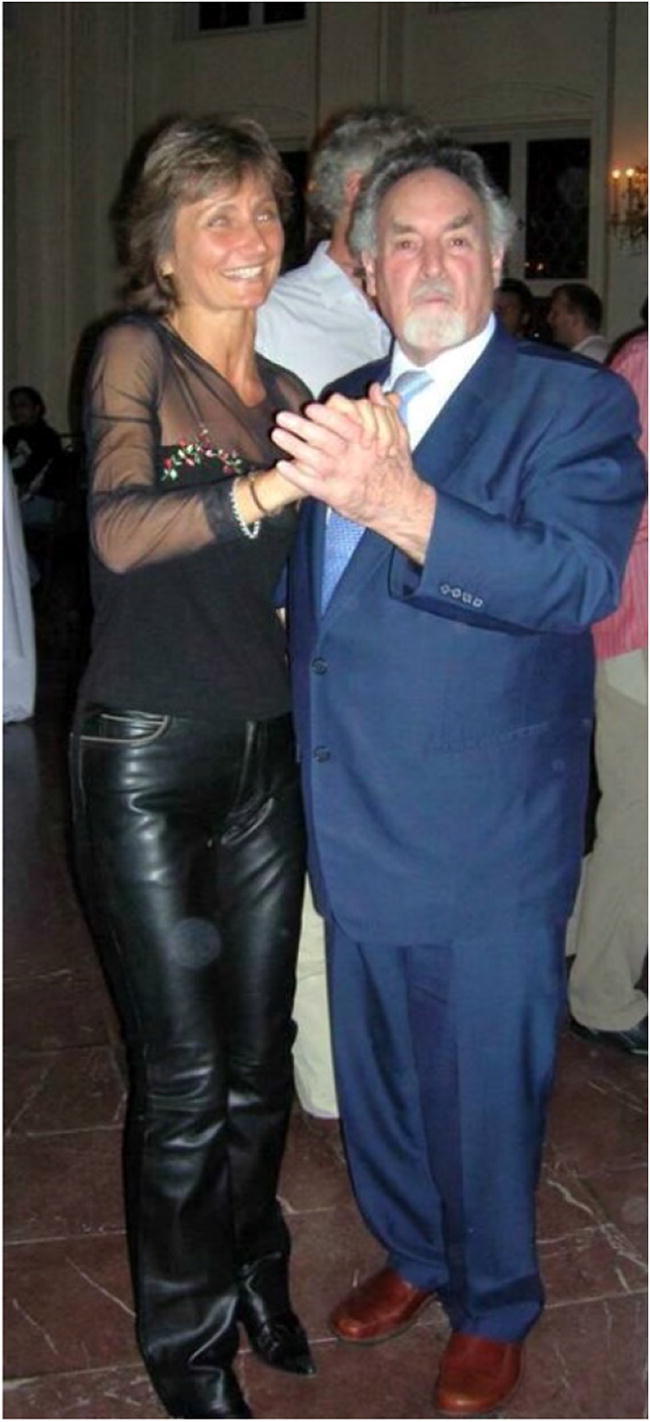
Cinzia Volonté and Geoff Burnstock dancing at the 2nd Joint Italian-German Purine Club Meeting 2007 (Leipzig, Germany)

The German Purine Club was officially founded in Madrid on the occasion of the international conference PURINES 2000 (organized by Maria Teresa Miras-Portugal, Jesús Pintor and Alexandre Ribeiro). It is crystal clear that the Club owes its existence to Geoffrey Burnstock’s seminal work and to his energetic support and encouragement (see Fig. 2). Herbert Zimmermann (Frankfurt) and Peter Illes (Leipzig) were the driving forces, and both were elected as the first Vice President and President, respectively. In 2008, Herbert Zimmermann became President (until 2015), with Peter Illes (2008–2010), Christa Müller (Bonn, 2010–2014) and Christian Lohr (Hamburg, 2014–2015) as Vice Presidents. Subsequently, Christa Müller took over as German Purine Club President, supported by Anke Schiedel (Bonn) and Christian Lohr as Vice Presidents.

The German Purine Club represents a loose association of scientists on all levels including students and advanced scientists active in this field in Germany. Researchers from other European countries, who do not have their own national Purine Club, are also welcome. Currently, it has 110 members. A newsletter is distributed on a regular basis to all club members. There are no membership fees.

The German-Italian/Italian-German Purine Club meetings initiated by our Italian colleagues were important catalysts for further strengthening the interactions, in particular also with our Italian colleagues. As described above, seven of these international meetings have taken place, alternately in Italy or in Germany, and in 2019, the meeting was combined with the First European Purine Meeting, which took place in Santiago de Compostela, Spain, hosted by M. T. Miras-Portugal.

### The Japanese Purine Club by Schuichi Koizumi, Yamanashi, Japan

**Fig. 3:**
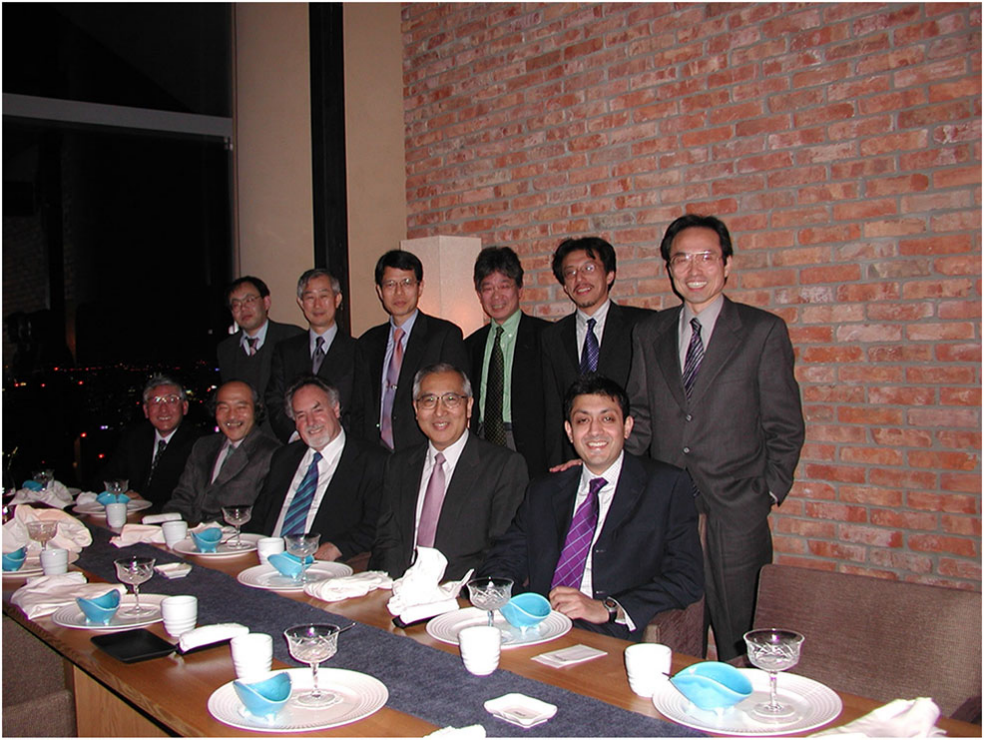
Welcome party of the 1st Japanese Purine Club Meeting (2003). From left in the front row: Taku Nagao, Yoichiro Kuroda, Geoff, Akio Sato, Baljid Kahkh. From left in the back row; Yoshinori Kawai, Shigeru Kageyama, Satoshi Kurihara, Fusao Kato, Schuichi Koizumi, Kazuhide Inoue

**Fig. 4:**
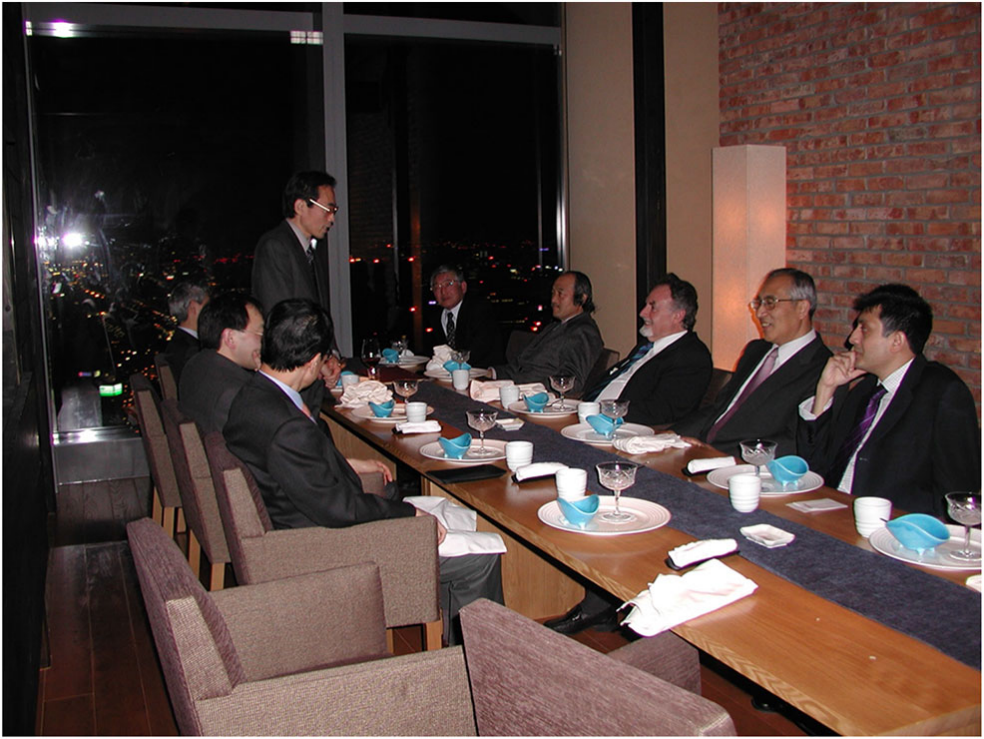
Welcome speech by Kazu (Prof. Inoue) in the welcome party of the 1st Japanese Purine Club. Standing, Kazu; from left in the back row: Taku Nagao, Yoichiro Kudoa, Geoff, Akio ato, Baljit Khahk

**Fig. 5:**
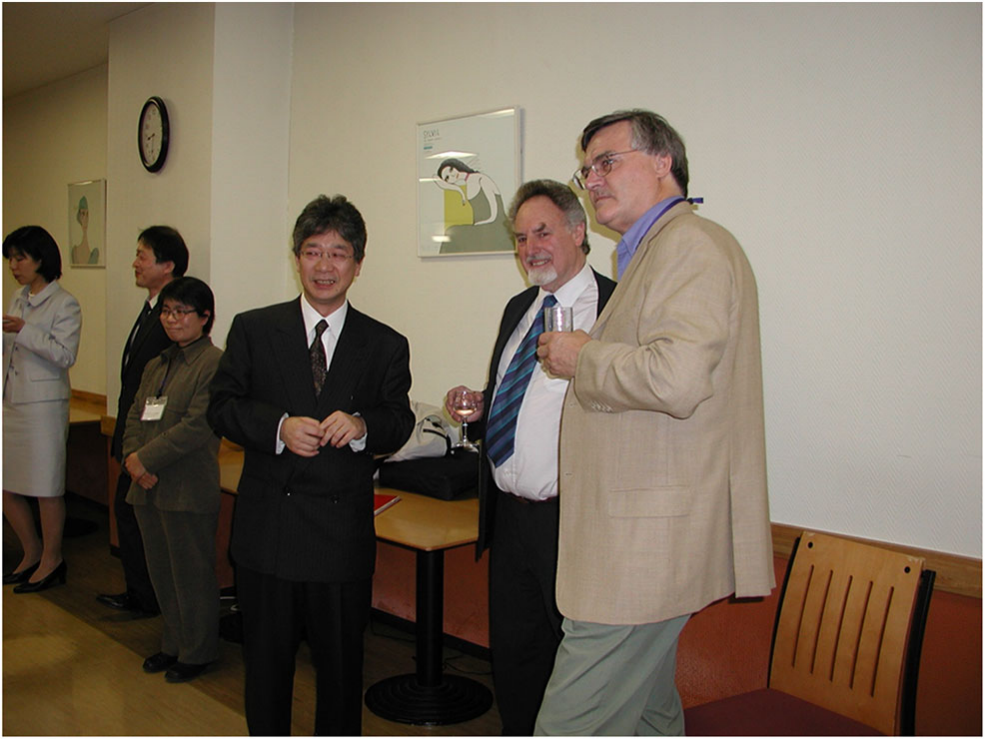
P2X receptor talk at the banquet of the 1st Japanese Purine Club. From left: Izumi Hide, Yoshihiro Nakata, Junko Kimura, Fusao Kato, Geoff, Alan North

The first recorded Purinergic Signalling Meeting in Japan took place in Nagasaki in 1996. After that, Prof. Kazuhide Inoue led purinergic signalling research, and founded the Japan Purine Club in 2003. The first President was Prof. Inoue, and the founding core members were Prof. Fusao Kato, Isao Matsuoka, Schuichi Koizumi and Makoto Tsuda. In that year, we invited Prof. Burnstock to the first meeting, and celebrated the founding of the Japanese Purine Club. It was an undoubtedly exciting, wonderful and memorable meeting (Figs. 3, 4, and 5). Since then, Japanese Purine Club meetings have been held almost every other year in Japan. Among them, Fukuoka Purines 2009 and Purines 2012 were held in Fukuoka with a large number of international guest speakers. Of course, Prof. Burnstock joined us for these meetings. He always supported and encouraged the Japanese Purine Club and was always a spiritual pillar of us. We are deeply saddened by the news of his passing away. We will not and cannot forget him, and now we would like to once again offer our truly heartfelt thanks for his support. With our gratitude and respect to Prof. Burnstock, we intend to further develop purinergic signalling research in Japan and the Japanese Purine Club. First, we are planning a memorial symposium for Prof. Burnstock. Since 2016, Prof. Schuichi Koizumi has taken over the running of the Japanese Purine Club.

### The Brazilian Purine Club: inspiring dream of purinergic signalling in South America by Henning Ulrich, Ana Maria O. Battastini, Ana L.M. Ventura, Robson Coutinho-Silva, Brazil

The foundation of the Brazilian Purine Club (BPC) in 2009 by Robson Coutinho-Silva, Ana Maria O. Battastini and Henning Ulrich followed from the desire of the Brazilian scientific community, interested in diverse aspects of purinergic signalling, to meet regularly and discuss their projects and results, in view of the positive results of the German and Italian Purine Clubs. The founders of the BPC were encouraged by Prof. Geoffrey Burnstock to gather Brazilian scientists and students, together with scientists from abroad, in annual meetings for discussing the latest advances in this area. This initiative, promoting new collaborations and networks, integrated Brazilian researchers and students in a scientific field which is rapidly expanding in Brazil and worldwide.

The first annual meeting, held in 2010, was honoured by the participation of Profs. Burnstock, Francesco Di Virgilio, Peter Illes, Rodrigo Cunha and other leaders of the field of purinergic signalling and was considered as excellent by the participants. Prof. Burnstock was particularly excited by the excellent research developed by Brazilian students and young scientists. This first meeting was followed by annual meetings joined by scientists from abroad. The high quality of the previous meetings, demonstrated by increasing number of participants and members of the society, encouraged us to organize the “Fifth Meeting of the BPC and Second International Congress on Purinergic Signalling in South America”. This congress also counted on the prestigious participation of Prof. Burnstock, and its excellent outcome resulted in the invitation for the BPC to hold the International Congress of all Purinergic Signalling Societies in Brazil in 2018. This congress, organized by Prof. Henning Ulrich, was opened by a video message from Prof. Burnstock, who was unable to travel abroad due to health limitations. The congress counted on the participation of more than 300 scientists and students from 25 countries of four continents, strengthening the quality, international insertion and recognition of the quality of Brazilian science, stimulating the exchange of ideas and promoting collaborations with researchers from abroad. A further congress was held in 2019, and the 2020 congress was postponed to the next year as consequence of the COVID-19 pandemics.

Prof. Burnstock became an Honorary Member of Brazilian Purine Club in recognition of his vast achievements in science and his supportive contribution to the establishment of the BPC. His friendship and continuous advises to Brazilian scientists and his encouragement to pursue science fostered the strength and excellence of research in Brazil and many other countries. He has inspired the dream of a dynamic scientific society of purinergic signalling in South America.

### The UK Purine Club by Allie Gartland and Leanne Stokes, UK

Despite the UK being the scientific home of Prof. Burnstock and a very active community of purinergic researchers, the establishment of a UK Purine Club was many years in the planning and a somewhat recent event. It was at the 8th International Symposium on Adenosine and Adenine Nucleotides held in Ferrara, Italy, in 2006 that the first conversations were held between myself, Prof. Alison Gartland and Prof. Geoff Burnstock about the lack of a UK Purine Club. We both felt given the large number of people at the Ferrara meeting from the UK that surely there were enough people to establish a club—but how exactly to do it! Geoff had the connections but not the energy (his words), and I had the energy but not the connections—so the perfect team was created! Together/between us, Geoff and I attended meetings of the Italian, German and Brazilian Purine Clubs (in some amazing locations I have to say) over the next few years to gather ideas about how to set up the club, the focus of the club and how keep it running. The UK Purine Club was eventually founded, and an inaugural meeting was held in Sheffield, in November 2009. Since then we have had annual meetings taking place in Nottingham, Cardiff and Norwich, Cambridge, Leicester and a joint meeting with the Italian club in Bristol.

The aim and focus of the UK Purine Club is to facilitate the bringing together of purine researchers in the UK and, beyond, to exchange scientific ideas. The club prioritizes giving early career researchers the platform to present their scientific work whilst at the same time the opportunity to network with some of the more established researchers in the field—something that Geoff was very passionate about—and said kept him “young”!

At the 10th anniversary of the establishment of the UK Purine Club, I stepped down as President and left the club in the capable hands of Dr. Leanne Stokes, who had this to say “Being part of the UK Purines Club has given me a sense of belonging, a sense of community and access to a network of scientists who are passionate about the same things as I am. It is an honour to be able to take the UK Purines Club forward to continue the shared community, to try to keep our membership growing and to provide opportunities for new collaborations and discussions. This is a fantastic support network for scientists at all stages of their career.”

### The Chinese Purine Club by Yong Tang, Chengdu, China

In the winter of 2011, Prof. Geoffrey Burnstock made a recommendation to Prof. Yong Tang (Chengdu University of Traditional Chinese Medicine, China) to establish a broad collaboration with purine scientists to test the purinergic hypothesis of acupuncture and move forward the purine story in China. Prof. Peter Illes (University of Leipzig, Germany) was proposed by Prof. Burnstock to work together with Prof. Yong Tang to submit a common proposal to Sino-German Centre (Beijing, China) for supporting the “1^st^ Sino-German Symposium on Purinergic Signalling, Pain and Acupuncture” in Chengdu between October 10 and15, 2012. After this meeting, the increasing number of funded projects, interested researchers, publications and the successful organization of the 2^nd^ Sino-German Symposia on Purinergic Signalling, Pain and Acupuncture held in Leipzig between September 26 and October 1, 2017, provided the occasion to set up the Chinese Purine Club. In 2018, the Chinese Purine Club was founded by co-chairs Yong Tang and Jiang-Fan Chen, with Geoffrey Burnstock and Peter Illes as honorary chairs.

The “1^st^ Chinese Purine Club meeting” was organized in Chengdu between October 10 and 12, 2018. Prof. Burnstock delivered the opening lecture at this meeting and also at the “1^st^ and 2^nd^ Sino-German Symposia on Purinergic Signalling, Pain and Acupuncture” (Chengdu, 2012 and Leipzig, 2017). Geoff also invited Prof. Yong Tang to join the Editorial Board of Purinergic Signalling in 2018 and to be an associate editor in November, 2019. After the foundation of Chinese Purine Club, Geoff continued to give great support and, together with Prof. Peter Illes and Yong Tang, proposed that the Chinese Purine Club organize the Euro-Asian Forum on Purinergic Signalling in Chengdu, China from 6 to 9 September 2020 (postponed to 2022 due to the COVID-19 emergency).

### The Australian and New Zealand Purine Club by Jennie M. E. Cederholm, Ronald Sluyter and Srdjan M. Vlajkovic, Australia and New Zealand

In November 2017, Geoffrey Burnstock moved back from London to Melbourne, Australia, and continued working as a Professorial Fellow at the University of Melbourne and an Honorary Professorial Fellow at the Florey Institute of Neuroscience and Mental Health. In July 2018, Prof. Burnstock contacted some of his purinergic signalling colleagues in Australia promoting the idea of establishing a local Purine Club. He became the Club’s first President, but also sought interest from “a young, energetic and ambitious scientist to take on the role of Vice President.” As a result, Dr. Jennie Cederholm (University of New South Wales) and Prof. Ronald Sluyter (University of Wollongong) were appointed as Co-Presidents. The Australia and New Zealand Purine Club (https://anzpurineclub.com/) was formally established in Spring 2018 [1]. Shortly after, Assoc. Prof. Srdjan Vlajkovic (University of Auckland) joined as the New Zealand Representative on the Executive Committee.

The inaugural meeting of the Australian and New Zealand Purine Club was held in May 2019 [2]. At this meeting, Prof. Burnstock delivered the opening address, providing an overview of purinergic signalling, past, present and future. This was the first opportunity for many early career researchers to hear and meet with Prof. Burnstock, a great teacher of science and life. Using his extensive reputation in the field, Geoffrey Burnstock was instrumental in establishing the Australian and New Zealand Purine Club. He brought together Australian and New Zealand purinergic researchers from a broad range of disciplines and attracted funding to support the inaugural meeting.

In July 2019, the Executive Committee was expanded to include Dr. Carolina De Moura Gubert (Florey Institute of Neuroscience and Mental Health) and student representative Reece Sophocleous (University of Wollongong). In Spring 2019, Prof. Burnstock shared his vision to strengthen purinergic research across the Asia-Pacific region proposing that the Australian and New Zealand Purine Club host a joint meeting with delegates of the China Purine Club. This meeting was organized for May 2020, but due to the COVID-19 pandemic, this meeting was postponed. The next Australian and New Zealand Purine Club meeting is scheduled for Autumn 2021, with the intention to continue regular meetings into the future. The club will continue to pursue its goals [1] including opportunities to engage with the China and Japanese Purine Clubs and the broader international purine community. Geoffrey Burnstock thus provided an ongoing legacy to current and future generations of purinergic researchers across Australia and New Zealand and beyond.

### The North American Purine Club by Kenneth A. Jacobson, Bethesda, USA

A North American Purine Club including members from both the USA and Canada was initiated in October 2009 with the President Ken Jacobson, Secretaries Jonathan Dranoff and Jean Sévigny, and the Organizing Committee members Luiz Belardinelli, José Boyer, Jiang-Fan Chen, Pamela Conley, Bruce Cronstein, Ken Harden, Ed Inscho, Joel Linden, Allen Moorman, Michael Rathbone, Katya Ravid, Simon Robson, Michail Sitkovsky, Gary Weisman, Connie Wilson and Jeff Zablocki. There is still an active Purine Club web presence on LinkedIn. There are 585 members. Ken Jacobson posts announcements about all of the international purine conferences, and there are also occasional postings about publications or job openings.
